# The adult congenital heart disease collaborative care model: Improving access with a care delivery innovation

**DOI:** 10.1016/j.ijcchd.2026.100709

**Published:** 2026-08-20

**Authors:** Anne S. Kemble Luo, Charlotte Park, Ramon Ruiz, Todd B. Seto, Stephen B. Kemble, Andras Bratincsak

**Affiliations:** a The Queen's Medical Center, Honolulu, HI, USA; b University of Hawaii, Honolulu, HI, USA; c Hawaii Pacific Health, Honolulu, HI, USA

**Keywords:** Adult congenital heart disease, Collaborative care, Access, Access gap, Care model, Care delivery

## Abstract

**Background:**

There is a critical nationwide access gap for the care of adults with congenital heart disease (ACHD). In Hawaii, where there are only 2 ACHD cardiologists serving the broader state-wide healthcare system, at most 10% of ACHD patients receive specialty care. Our goal was to 1) design and implement a novel program structured to provide high-quality ACHD care to a large population of patients by a limited number of ACHD specialists; and 2) evaluate the preliminary impact of this program on ACHD specialty care clinic volume.

**Methods:**

We built a novel care delivery model wherein the University of Washington AIMS Psychiatric Collaborative Care Model (CoCM) is adapted to expand the reach of ACHD specialists via team-based care coordination and integration of adult cardiologists and primary care providers. To evaluate the impact on access, we compared clinic volume during the pilot implementation phase in April - May 2026, to the volume of patients seen during 2023.

**Results:**

Within 5 months of implementation, the ACHD CoCM average weekly clinic volume was 19.6 patients per 0.2 FTE ACHD cardiologist, compared to the traditional care model baseline of 10.5 ACHD patients.

**Conclusions:**

We launched a novel ACHD CoCM to improve access to high-quality ACHD care using carefully orchestrated care coordination and integration of primary treating providers, with preliminary data suggesting a ∼2-fold increase in specialty care clinic volume. We estimate with refinements this could ultimately expand each ACHD cardiologist's panel capacity by 3.3 times compared to a traditional care model.

## Highlights

• To address the congenital heart care gap, we need to rethink how care is delivered.
• We developed a collaborative care model for congenital heart disease.
• A novel adult congenital heart disease care model increases access 2-fold.

## 1 Introduction

By conservative projections >0.6% of the adult population is living with congenital heart disease, with an estimated prevalence of 1.69 million in the US in 2024 [1]. There is a severe nationwide shortage of Adult Congenital Heart Disease (ACHD) specialists to care for this growing population of complex, high-risk patients​. Solving this access gap will require a multifaceted strategy including training more specialists and advanced practice providers, incorporating telehealth and information technology, and reducing insurance barriers [1]. In addition, we believe it is necessary to rethink how care is delivered.

### 1.1 Hawaii's access gap

Based on data from The Queen's Health Systems and Hawaii Pacific Health, the two largest healthcare systems in Hawaii, only 10% of patients with an ICD-10 diagnosis of an ACHD condition received care from an ACHD specialist between 2023 and 2025. Further, among patients hospitalized with an associated ACHD diagnosis during this period, only 6% had received any preventive specialty care (defined as outpatient care by an ACHD specialist preceding the admission by 1-3 years).

Like other rural areas in the United States, Hawaii's care gap is compounded by geographic distance. With 7 inhabited islands, the 2 specialty-trained ACHD physicians serving the broader statewide healthcare system are located on the predominantly urban island of Oahu. As is common outside of ACHD Comprehensive Care Centers, their clinical practice is divided between ACHD and adult or pediatric cardiology. The 2024 US Census indicates neighbor islands are home to ∼30% of the state's population [2], but account for only 15% of those cared for by our ACHD program. This reflects an even more severe access gap for neighbor island communities geographically separated from subspecialty care by ocean.

To address this access gap, we propose a novel approach to cardiac care wherein the University of Washington AIMS model of Psychiatric Collaborative Care is adapted to expand the reach of ACHD specialists via team-based care coordination. Our goal was to 1) design and implement this program; and 2) evaluate its preliminary impact on ACHD specialty care clinic volume.

## 2 Methods

Our model is based on the psychiatry Collaborative Care Model (CoCM) at The Queen's Medical Center, which follows the University of Washington AIMS model and has been implemented in Hawaii since 2017, using a unique payment structure with pooled direct funding from Hawaii's insurance payors. The Psychiatry CoCM tracks well-defined populations in a registry to ensure clinical outcomes are met by integrating care managers and primary care physicians with oversight by a psychiatrist​ [3]. We performed a literature review to understand how CoCMs are applied to different populations and worked with our internal team and IT department to adapt the program for ACHD. To evaluate the impact on ACHD specialty care access, we defined clinic volume as the weekly number of patients directly evaluated by the CoCM team during the pilot implementation phase (April – May 2026). We compared this to the weekly volume of patients seen during 2023 (the most recent year the “traditional” ambulatory ACHD clinic was fully available prior to preparations to transition care models).

## 3 Results

The ACHD CoCM is a team-based care model involving the following roles and responsibilities.

i. An ACHD cardiologist establishes an individualized surveillance plan for each patient, reviews test results and clinical status updates, and adjusts care plans accordingly. In-person visits are mainly focused on patients with higher complexity conditions or requiring a change in care plan or peri-procedural care.
ii. ACHD care managers use an Epic-synchronized REDCap registry to identify when patients are due for recommended testing and follow-up. The REDCap registry imports data directly from the electronic health records to improve efficiency. Care managers gather data directly from patients and their charts for physician review, coordinate testing, provide health maintenance counseling, and facilitate communication among the patient and care team. They tailor follow-up to patient acuity and needs, ensuring timely access to ACHD specialists when needed.
iii. A primary treating clinician (a primary care physician, adult cardiologist, or advanced practice provider) partners with an ACHD cardiologist and care manager to provide surveillance care. Routine office-based visits and medication adjustments are primarily delivered by the primary treating clinician, with communication and guidance from the ACHD team.

This care model reimagines traditional follow-up. Rather than seeing every patient annually, the ACHD specialist focuses on new referrals, patients needing specialized counseling or changes in management, and oversight of care. At the same time, the frequency of patient touchpoints increases through care manager outreach, telehealth, phone calls, and remote (non-visit-based) consultations. This care model is designed to reduce lapses in care by improving access, re-engaging patients lost to follow-up, and supporting patients and local providers, including those in geographically remote communities.

Currently, 305 patients are enrolled in the ACHD CoCM program, run by a part-time (0.2 FTE) ACHD board-certified cardiologist and 4 part-time care managers who are social workers (0.5 FTE total) since January 2026. The ACHD CoCM average weekly clinic volume after 5 months was 19.6 patients per 0.2 FTE ACHD cardiologist, compared to the 2023 baseline of 10.5 ACHD patients.

## 4 Discussion

**Fig. 1:**
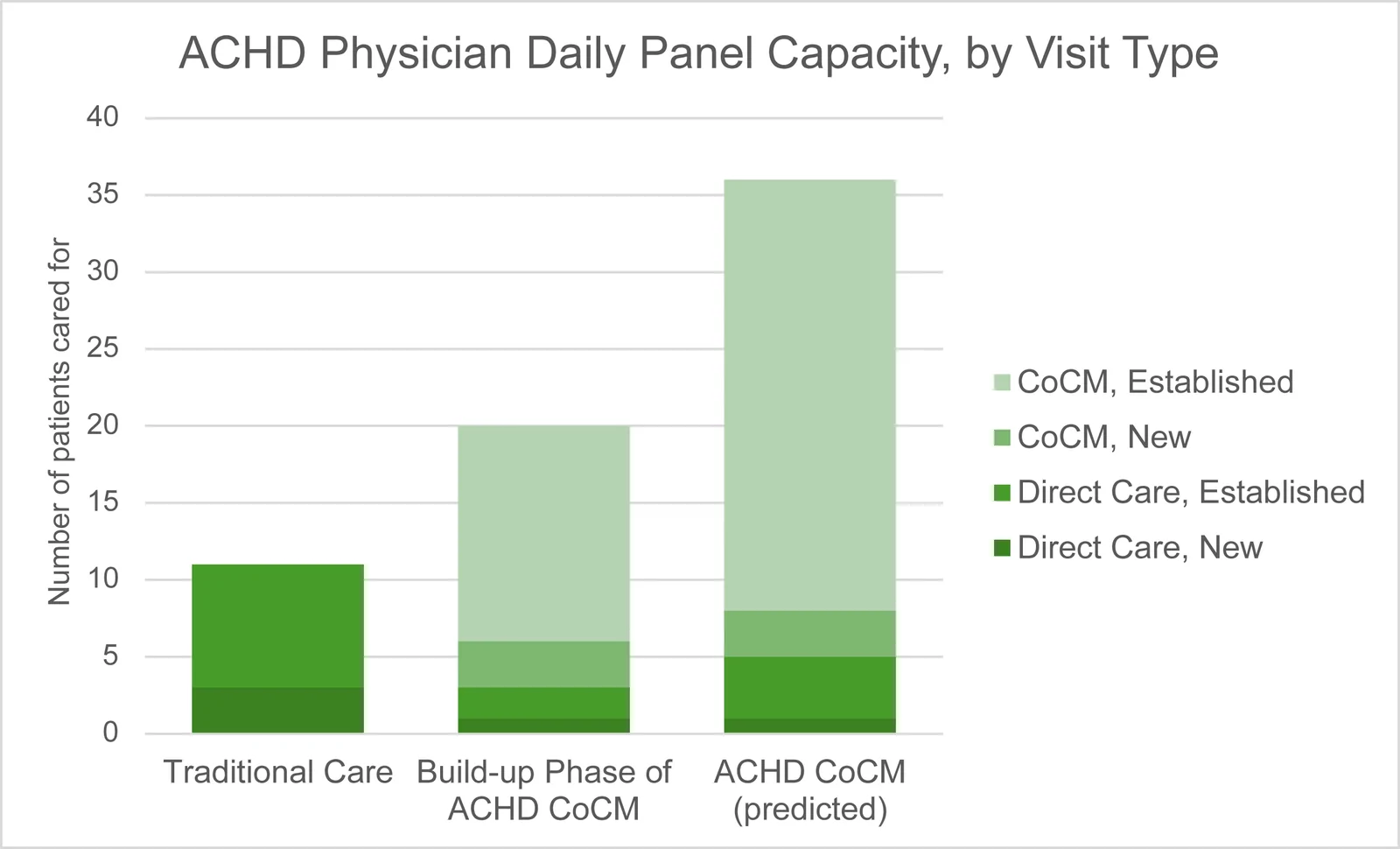
Projected daily panel capacity for an ACHD physician specialist in a traditional care model and ACHD CoCM, demonstrating the current (build-up phase) and predicted expansion in capacity. Visit types are indicated by shade. Abbreviations: ACHD Adult Congenital Heart Disease, CoCM Collaborative Care Model.

We developed and implemented a novel ACHD CoCM to improve access to high quality ACHD care using carefully orchestrated care coordination and integration of general cardiologists and primary care providers, with preliminary data suggesting a ∼2-fold increase in specialty care clinic volume. In the early (build-up) phase of the ACHD Collaborative Care Model, our clinic volume data supports the following projection (Fig. 1).

i. Initially, for each 0.2 FTE module (defined as 8 h per week of office-based care), an ACHD specialist can provide direct care for approximately 1 new and 2 established patient visits, and 3 new and 14 established remote evaluations. Care is provided for ∼20 patients.

Although our initial data is encouraging, we plan to further study the impact of the care model on clinic volume and other markers of access and quality as the program matures. Based on the observed average time spent on each task type versus the time allotments we believe are achievable with optimization and gained experience, we speculate that the clinic volume demonstrated thus far underestimates the potential of the care model. For instance, with optimization of workflows and IT refinements, our team anticipates increased efficiency, particularly in remote care. We predict that the following clinic volumes are feasible.

ii. For each 0.2 FTE module weekly, an ACHD specialist may be able to provide approximately 1 new (1 h) and 4 established (2 h) direct care visits, and 3 new (1.5 h) and 28 established (2.5 h) remote evaluations. It would be feasible to provide care for ∼36 patients (3.3 times greater capacity) while still leaving time for administrative or other care coordination work.

### 4.1 Funding strategy

ACHD care is primarily preventive, and therefore cost-saving [4]. However, the time required to care for these complex patients is not reimbursed in a sustainable way by the existing fee-for-service payment structure [5]. We are working toward a sustainable funding structure in collaboration with Hawaii's insurance providers for value-based care, mirroring the Hawaii Psychiatry CoCM. Our institution's Psychiatry CoCM internal data demonstrates reduction in administrative costs by 20-25% versus fee-for-service, improved access with a patient engagement rate of 78% of referrals, improved quality via access to subspecialist care, and reduced costs via reductions in inappropriate utilization and readmissions​. A similar ACHD model should also achieve favorable results with improvements in quality, reach, and patient outcomes, while reducing costs.

## 5 Study limitations

There are several limitations to our study. First, our findings are based on early, short-term data and primarily use clinic volume as a proxy for access. Second, while increased volume is encouraging, it does not fully capture access, quality or patient outcomes. Third, the pre-post design of our study does not establish causality. Finally, our findings reflect the experience of a single health system in Hawaii and may not be generalizable to other settings. For example, Hawaii's geographical isolation, patient population, and workforce are distinctive, and in particular the funding strategy thus far is unique, which may influence both the feasibility and impact of the model.

## 6 Conclusions

We have developed a collaborative care model for ACHD in Hawaii to improve access to care, reduce costs, and improve the capacity of the limited number of ACHD specialists. We are piloting an ACHD CoCM program and collecting data, with hopes to replace standard fee-for-service reimbursement with pooled direct funding. As an early implementation study, these findings suggest an encouraging impact of the care model on clinic volume as a proxy for access, however the findings should be considered preliminary. Additional studies are needed to evaluate the model's reproducibility, and its effects on access, quality of care, patient outcomes, and healthcare utilization. If successful, this may be a highly effective and financially sustainable means of addressing the severe access gaps facing adults living with congenital heart disease nationally. Further work is required to assess the reach, adoption, implementation, maintenance and effectiveness of this program, and alternative funding strategies should also be explored.

## CRediT authorship contribution statement

**Anne S. Kemble Luo:** Writing – original draft, Methodology, Funding acquisition, Conceptualization. **Charlotte Park:** Writing – original draft, Investigation. **Ramon Ruiz:** Writing – review & editing, Investigation. **Todd B. Seto:** Visualization, Supervision. **Stephen B. Kemble:** Writing – review & editing, Methodology, Conceptualization. **Andras Bratincsak:** Writing – review & editing, Supervision, Methodology, Conceptualization.

## Acknowledgement of grant support

1 10.13039/100005485American College of Cardiology 2025 Chapter/Section Grant, (ACC, Washington, DC)
2 UHA Health Insurance Program Quality Grant (UHA, Honolulu, HI)

## Declaration of competing interests

The authors declare the following financial interests/personal relationships which may be considered as potential competing interests: Anne Kemble Luo reports financial support was provided by American College of Cardiology. Anne Kemble Luo reports financial support was provided by University Health Alliance. Charlotte Park reports financial support was provided by American College of Cardiology. If there are other authors, they declare that they have no known competing financial interests or personal relationships that could have appeared to influence the work reported in this paper.
